# Phase Field Simulation of the Effect of Second Phase Particles with Different Orientations on the Microstructure of Magnesium Alloys

**DOI:** 10.3390/ma16186329

**Published:** 2023-09-21

**Authors:** Yan Wu, Jinlin Xiong, Shuo Wang, Junsheng Yang, Mingtao Wang

**Affiliations:** 1 School of Mechanical Engineering, Wuhan Polytechnic University, Wuhan 430023, China; 2 School of Materials Science and Engineering, Northeastern University, Shenyang 110819, China

**Keywords:** second phase particles, AZ31 Mg alloy, phase field models, microstructure refinement

## Abstract

In this study, the phase field method has been used to study the effect of second phase particles with different shapes and different orientations on the grain growth of AZ31 magnesium alloy, after annealing at 350 °C for 100 min. The results show that the shape of the second phase particles would have an effect on the grain growth; the refinement effect of elliptical particles and rod-shaped particles was similar, and better than the spherical particles; the spatial arrangement direction of the second phase particles had no significant effect on the grain growth. On the other hand, when the microstructure of AZ31 magnesium alloy contained second phase particles with different shapes, the effect of mixing different shapes of second phase particles on the grain refinement was enhanced gradually with the decrease im the volume fraction of spherical particles.

## 1. Introduction

As environmental pollution and energy shortages have become more and more serious, society’s call for energy conservation and environmental protection has increased dramatically [1]. AZ31 magnesium alloy [2] has received a lot of attention from researchers due to its low density, superior vibration and noise damping performance, excellent electromagnetic shielding performance, non-pollution, easy recycling and other characteristics, which has an unparalleled and important role in promoting the national strategy of carbon neutrality and carbon peaking [3,4].

However, when AZ31 magnesium alloy is used directly as a structural material, it has been limited by its crystal structure of HCP, which has only one accessible slip surface at room temperature, so only three dense directions (three slip systems) could induce plastic deformation, which results in insufficient strength, plasticity, wear resistance and especially high-temperature performance, and limits its applications [5,6]. To solve these problems, researchers often introduce second-phase particles to achieve fine grains to improve the strength of the alloys [7,8,9,10]. The microstructure of AZ31 magnesium alloy consists of a large number of equiaxed grains, and the size of equiaxed grains directly determines the strength of the organization; the more equiaxed grains, the finer the grains, and the greater the strength of the material. As the grain boundaries have higher dislocation density and free energy than the inner parts of the grains, they may absorb and disperse the dislocations and stresses generated when plastic deformation occurs. Therefore, grain refinement could disperse more stress at grain boundaries, thereby improving the strength, plasticity and toughness of the material. Moreover, there are numerous grain boundaries within the microstructures with fine grains, which are even more tortuous. When microcracks appear under action of external forces, these tortuous grain boundaries will hinder the crack growth, effectively improving the fracture toughness and fatigue life of materials. Therefore, the mechanical properties and durability of the material can be effectively improved by achieving grain refinement. This is well demonstrated in the literature [11,12,13].

It has been shown that conducting material research solely through experimental methods not only incurs high costs, but also in many cases, leads to a lack of information on microstructure evolution mechanisms, and it cannot explain conflicting data, resulting in the inability to identify true patterns [14]. The advantages of numerical simulation techniques have been gradually highlighted with the progress of science and technology, and the use of computer technology to study modern science has become a proven scientific aid [15]. Many computational models have been used in the material research, such as the Front tracking models [16,17,18], Monte Carlo models [19,20,21,22], and phase field simulations [23,24], etc.

In this study, a multiorder parametric phase field model has been used to realize simulations at real spatial and temporal scales, and the effects of second phase particles with different shapes, spatial arrangement directions, and mixed shapes on the grain growth of AZ31 magnesium alloy have been systematically studied at the annealing temperature of 350 °C for 100 min. The results of the study could provide an important academic reference for the in-depth investigation of the mechanism and law of the influence of second-phase particles on grain growth.

## 2. Model Description

The phase field model [25] constructs phase field equations to describe the dynamics evolution of the systems, considering the comprehensive effects of ordering potential and thermodynamic driving force. The models could describe diffuse interfaces by introducing the continuously varying order parameters, in order to avoid complex mathematical problems [26]. The equations have been called the Allen–Cahn equation and Cahn–Hilliard diffusion, as follows [23]:

(1) $$\frac{\partial \eta_{p}(r,t)}{\partial t}=-L\frac{\delta F}{\delta \eta_{p}(r,t)}\left(p=1,2,3,\ldots ,n\right)$$

(2) $$\frac{\partial c(r,t)}{\partial t}=M\nabla^{2}\frac{\delta F}{\delta c(r,t)}$$

where *L* is the interfacial mobility coefficient; *M* is the diffusion coefficient; *t* is the evolution time; r is the position; in order to describe the microstructure and different orientations of grains of polycrystalline materials, a series of continuous field variables are selected: *η*_1_(**r**, *t*), *η*_2_(**r***, t*), ……*η_p_*(**r***, t*), *η_n_*(*p* = 1,……*n*) are called the orientation field variables. They are spatially continuous, and used to distinguish different orientations of grains. The orientation field variable is defined as follows: in the grain labeled as *η*_1,_ the value of *η*_1_ is 1, then the value of other *η_p_*(*p* ≠ 1) is 0. When passing through grain *η*_1_ and its adjacent grain boundary range, the value of *η*_1_ continuously changes from 1 to 0. *n* is the number of grain orientations. In theory, the larger the value of *n*, the better, and it is set to 32 in this system as suggested in the literature [23]; *c*(**r**, *t*) is the concentration field variable, representing the concentration at different locations and time in the microstructure. *F* is the total free energy; it can be expressed as a function of field variables *η_p_*(**r**, *t*) and *c*(**r**, *t*). In this study, it is expressed as follows [23]:

(3) $$F=\int_{V}\left[\frac{K_{2}}{2}{\sum_{p=1}^{n}\left(\nabla \eta_{p}(r,t)\right)}^{2}+f_{0}(c,\eta_{1},\eta_{2},\ldots ,\eta_{p})\right]dr$$

where *K*_2_ is the gradient coefficient; *f*_0_ is the local free energy density function.

The work [23] suggests the introduction of a visual function in the construction of the free energy density function to describe the effects of second phase particles on the grain evolution.

(4) $$\varphi (r)=\Phi (r)\sum_{p=1}^{n}\eta_{p}^{2}(r)$$

where Φ is used to describe the particle distribution, and is taken as 1 when it is located at the second phase particle, otherwise it is 0.

The expression of the local free energy density function *f*_0_ [23] in this model is:

(5) $$\begin{array}{l} f(c,\eta_{1},\eta_{2},\ldots ,\eta_{p})=A+\frac{A_{1}}{2}{\left(c(r,t)-c_{l}\right)}^{2}+\frac{A_{2}}{4}{\left(c(r,t)-c_{l}\right)}^{4}-\frac{B_{1}}{2}{\left(c(r,t)-c_{l}\right)}^{2}\sum_{p=1}^{n}\eta_{p}^{2}\\ +\frac{B_{2}}{4}\sum_{p=1}^{n}\eta_{p}^{4}+\frac{K_{1}}{2}\sum_{p=1}^{n}\sum_{q\neq p}^{n}\eta_{p}^{2}\eta_{q}^{2}+\Phi \sum_{p=1}^{n}\eta_{p}^{2}(r) \end{array}$$

where *c*(**r**, *t*) is the concentration of Al; *c_l_* is the concentration content at the lowest point on the free energy concentration curve at a specific temperature; *A*, *A*_1_ and *A*_2_ are the constants associated with the free energy of the system; *B*_1_ and *B*_2_ are the coefficients; *K*_1_ is the coupling term coefficient between *η_p_* and *η_q_*.

In order to visually display the microstructure represented by orientation field variables, the following function is defined [24]:

(6) $$\omega (r)=\sum_{p=1}^{n}\eta_{p}^{2}(r)$$

Through calculation, it could be concluded that the function ω(**r**) took a value of 1.0 within the grain, and a relatively small value at the grain boundaries.

If ω(**r**) is represented as black with small values and white with large values, the gray scale (i.e., grayscale) is used to draw the pictures, so in the obtained microstructure, the bright parts are grains, while the black lines represent grain boundaries, which is similar to the microstructure observed by an optical microscope, making it easier to compare the simulated microstructure with the actual observed microstructure of alloys.

In this study, the grain boundary range has been chosen according to the reference [23]; it is considered that the grain boundaries are diffusion interfaces with a certain thickness. AZ31 magnesium alloys have been used as the simulated materials, and the annealing temperature is 350 °C. The simulation system is a two-dimensional system. Assuming each grid size *dx* is 0.293 μm, and there are 512 × 512 grid cells, the total area of the simulated area is 150 μm × 150 μm. The time step is selected as 0.3 s. Since the recrystallization nucleation is very complex, it is treated by the phenomenological method; the average simulation area 4 dx × 4 dx is used as the initial nucleation area, so the radius of the grain nucleus in each unit is a random value between zero and two grid cells. The other parameters in the simulations are set as [23]: *c*_1_ = 0.2, *A*_0_ = −25.01 kJ mol^−1^, *A*_1_ = 22.02 kJ mol^−1^, *A*_2_ = 18.30 kJ mol^−1^, *B*_1_ = 3.54 kJ mol^−1^, *B*_2_ = 92.86 kJ mol^−1^, *K*_1_ = 141.24 kJ mol^−1^, *K*_2_ = 35.37 J m^2^ mol^−1^, and the interfacial mobility coefficient *L* = 1.15 × 10^−2^ mol Js^−1^.

## 3. Simulation Results

### 3.1. Different Shapes of Second Phase Particles

In the current experiments [27], in order to improve the mechanical properties of AZ31 magnesium alloy, researchers added Ca, Sr, Ce and other alloying elements to the AZ31 magnesium alloy matrix to prepare second-phase particles by the in situ synthesis method, and it was found that the shapes of the particles synthesized in situ showed spherical, elliptical and rod-like shapes. Therefore, the effects of the shapes of the particles have been systematically investigated.

**Figure 1:**
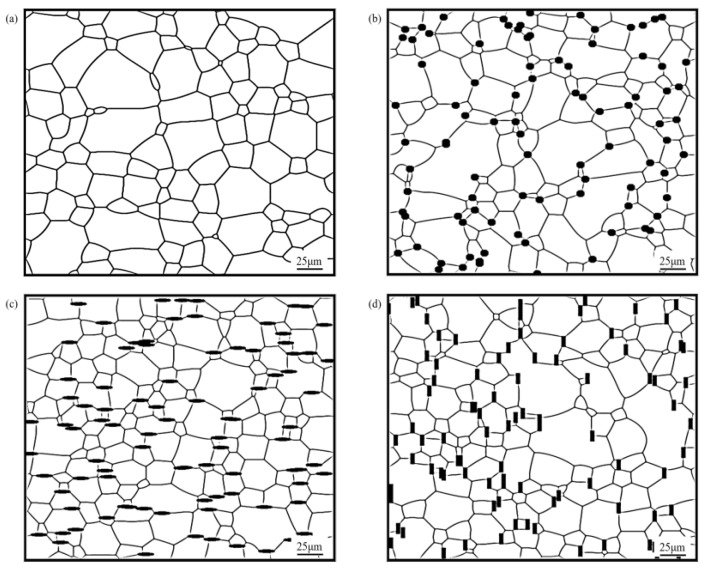
Microstructure of AZ31 magnesium alloy containing different shaped particles at annealing temperature of 350 °C and annealing time of 100 min. (**a**) AZ31 magnesium alloy matrix; (**b**) containing spherical particles; (**c**) containing elliptical particles; (**d**) containing rod-shaped particles.

In the study, different shapes of particles have been set in the models, such as circular particles with particle radius *r* = 2 μm, elliptical particles with a long semi-axis *a*_1_ = 4 μm and short semi-axis *b*_1_ = 1 μm, and rod-shaped particles with length *a*_2_ = 6 μm and width *b*_2_ = 2 μm. The effects of different particle shapes on the grain growth of Mg alloy were investigated at the annealing temperature of 350 °C, when the second phase particle volume fraction was *f* = 5%, with annealing for 100 min. The microstructure obtained is shown in Figure 1.

It can be observed from Figure 1 that the corresponding microstructures have been refined when containing second phase particles compared to the AZ31 magnesium alloy matrix. The spherical particles were mostly distributed on grain boundaries tending to trigonal grain boundaries, which is in accordance with the experimental results in the literature [27]. This is due to the fact that the bonding surface between different grains generates certain interfacial energy in the single-phase polycrystalline system, and when the spherical particles are located at grain boundaries, the total area of the boundaries will be reduced, resulting in a reduction in the interfacial energy, while when the spherical particles are located at trigonal boundaries, the maximum interfacial energy reduction effect could be achieved, which is consistent with the system evolution trend. In addition, elliptical and rod-shaped particles were located at the boundaries, and the final alignment directions were all along the grain boundary direction.

It could be found that the sizes of the grains near the particle were limited, while the sizes of the grains located far from the particle were relatively larger. This was because of the formation of a deformation zone in the matrix, which stored more energy and led to an increase in the nucleation efficiency, which could promote thermal recrystallization, thus promoting grain size homogenization and regularization during evolution.

**Figure 2:**
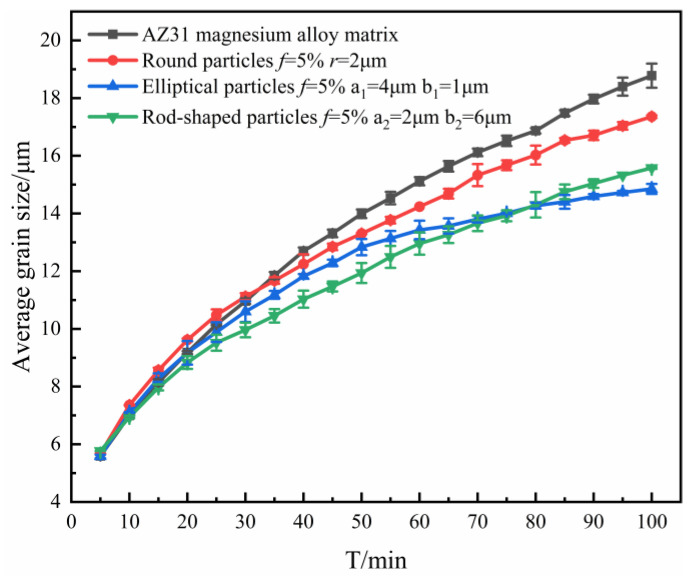
Variation of average grain size with time when containing particles of different shapes.

The curve corresponding to the average size variation of grains with annealing time was plotted, as shown in Figure 2.

From Figure 2, it could be found that the average grain size *R*_ave_ gradually increased. This is because, as the annealing time increased, the grains began to grow and fused to form larger grains, resulting in an increase in the average grain size. However, after the grains reached a certain size, the average grain size started to stabilize due to the prolonged presence of interfacial defects and energy, which limited the growth of new grains, and the grains started to compete with each other for growth.

In the early stage of grain growth (annealing time t < 10 min), the corresponding grain average size curves almost coincided, and as the evolution proceeded, differences gradually appeared because of the different shapes of particles, and the average grain size curves started to show different growth states. The reason for this phenomenon is that in the early stage of grain growth, the radius of curvature of the grain boundaries was large and the number of grains located on the grain boundaries was small, which resulted in the growth driving force of the grains being much larger than the pinning force acting on grain boundaries. As the evolution proceeded, more and more second phase particles were located on the grain boundaries, and then the pinning force of the particles on the grain boundaries was gradually increased. Moreover, the grain boundaries were gradually flattened, the radius of curvature gradually decreased, and the growth driving force gradually decreased. As a result, the grain growth curves showed a difference in later stages, which was mainly influenced by the particles. This phenomenon also indicates that the grain growth was mainly controlled by the migration of grain boundaries at the early stages of growth, and mainly influenced by the particles at the later stages, reflecting that the pegging effect of the particles on the grain boundaries was a process that gradually increased with the evolution time.

When different shapes of particles with *f* = 5% were added, the effects of elliptical and rod-shaped particles on the average size of grains were greater than those of spherical particles. This was due to the fact that the contact between elliptical and bar-shaped particles and grain boundaries was linear, and the contact area with grain boundaries was large, so the inhibition of grain boundary evolution and grain growth was stronger. In addition, compared with such equiaxed particles as circles, non-axial particles such as ellipses and rods had poor spatial symmetry and stronger hindrance in the direction perpendicular to their orientation, which caused the grain boundaries to show obvious directionality, making ellipses and rods come into contact with the grain boundaries in parallel. Therefore, the inhibition effect produced by elliptical, rod-shaped particles during grain growth was stronger than in the case of spherical particles. These results are in agreement with the suppression of the simulation results in the literature [28] and with the results derived from physical models [29,30,31].

### 3.2. Effect of Spatial Arrangement Direction of Second Phase Particles

In the study of the effect of the shape of the particles, it was found that non-isometric second phase particles such as ellipsoidal and rod-shaped particles have certain spatial arrangement directions in space [27]. Therefore, in this study, a simulation of the influence of non-equiaxial second phase particles’ spatial arrangement directions on the microstructure of AZ31 magnesium alloy was carried out with elliptical particles as an example.

**Figure 3:**
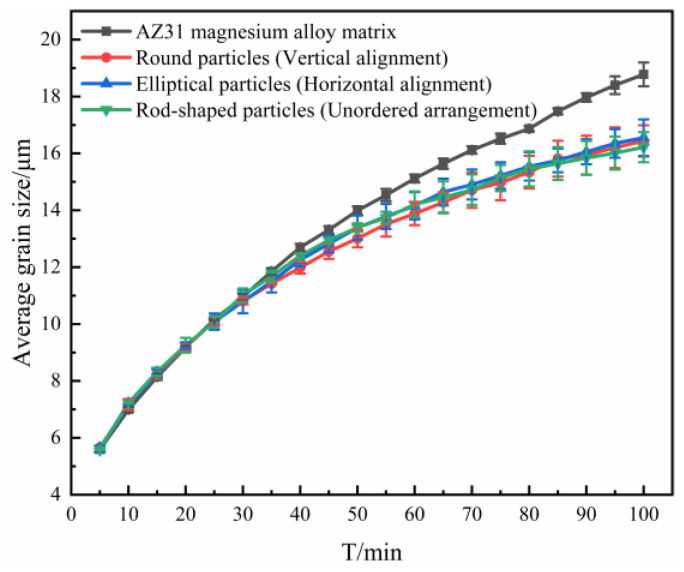
Curve of the average grain size with time when containing elliptical particles with different alignment directions.

It can be found from Figure 3 that the grains’ average size curves almost coincide exactly, corresponding to different particle spatial arrangement orientations. For this reason, it could be concluded that the spatial arrangement direction of the non-isometric second phase particles has no effect on average grain size. The reason for this result is due that, when the grains grew into equiaxed grains, ambiguity occurred in the direction of distribution of non-equiaxed grains. That is, the second phase particles with different alignment directions became indistinguishable under microscopic conditions.

### 3.3. Effect of Mixing Shapes of Particles

In view of the actual process, the second phase particle shapes in the system mostly existed in a mixture of different shapes [27]. For this reason, the study of the influence of different shapes of particles on the microstructure of AZ31 magnesium alloy was carried out.

**Figure 4:**
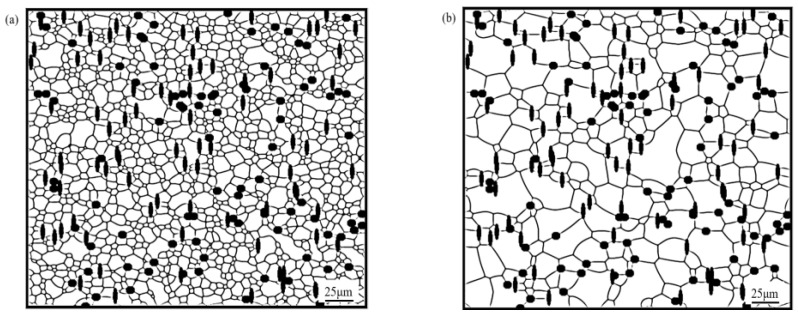

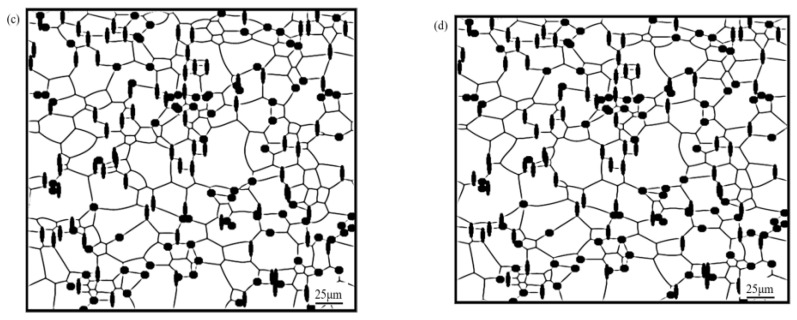
Microstructure evolution with time of AZ31 magnesium alloy containing equal volume fractions of mixed second phase particles of different shapes: (**a**) *t* = 5 min; (**b**) *t* = 40 min; (**c**) *t* = 70 min; (**d**) *t* = 100 min.

The simulation study was conducted at a 350 °C annealing temperature by adding spherical and elliptical mixed second phase particles with the volume fraction *f*_1_ = 5% and *f*_2_ = 5 and the size *r*_1_ = 2 μm, *a*_1_ = 4 μm and *b*_1_ = 1 μm, to AZ31 magnesium alloy. The simulated results are shown in Figure 4.

**Figure 5:**
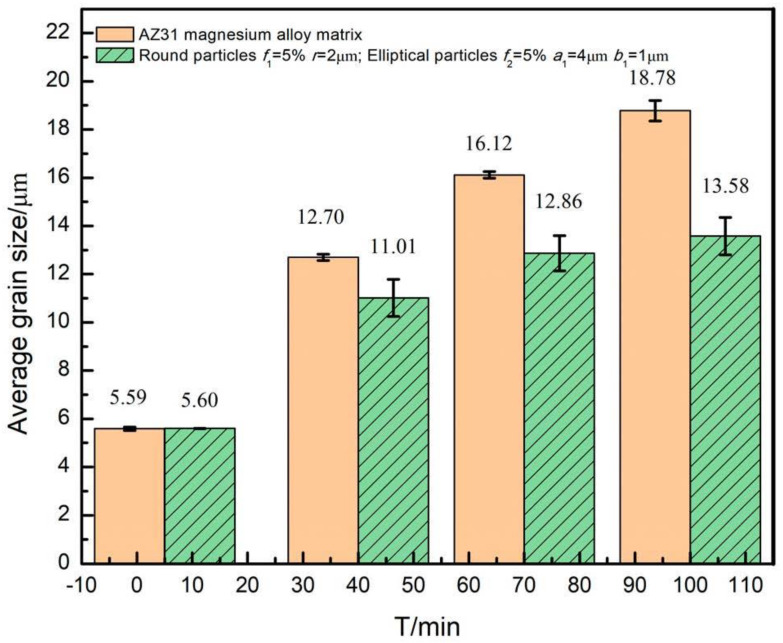
Diagram of the average grain size with time when containing spherical and elliptical mixed second phase particles.

From Figure 4, it could be observed that the second phase particles were diffusely distributed in the matrix, spherical particles tended to be located at the trigonal grain boundaries, elliptical particles were arranged along the grain boundaries, and the distribution of particles was consistent with the case of single-shaped particles, while the grain growth trend was also consistent, which was gradual growth until stabilization. The specific grain average size variations are shown in Figure 5.

It can be observed from Figure 5 that the difference in average grain size gradually increased in both cases, and the growth rate decreased until it became stable when containing mixed shapes of particles.

**Figure 6:**
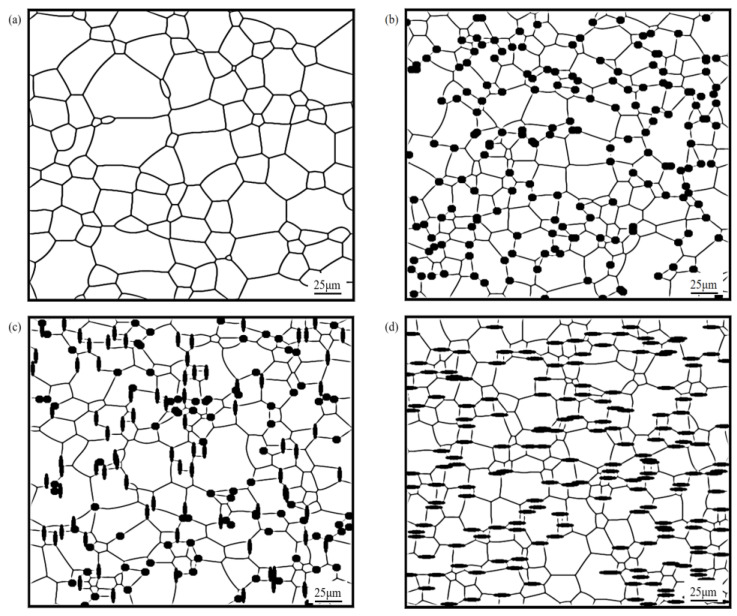
Microstructure diagram corresponding to an annealing temperature of 350 °C and annealing time *t* = 100 min: (**a**) AZ31 magnesium alloy substrate; (**b**) spherical particles *f* = 10%, *r* = 2 μm; (**c**) spherical and elliptical mixed particles *f*_1_ = 5%, *r* = 2 μm and *f*_2_ = 5%, *a*_1_ = 4 μm and *b*_1_ = 1 μm; (**d**) elliptical particles *f* = 10%, *a*_1_ = 4 μm and *b*_1_ = 1 μm.

The case of a mixture of spherical and elliptical particles is compared with the corresponding single-shape case, as shown in Figure 6.

By observing Figure 6, it could be found that the second phase particles without spherical shapes have the most obvious grain refinement effects.

**Figure 7:**
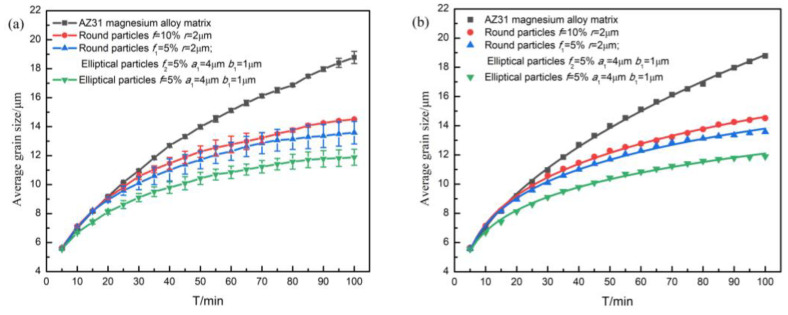
simulated results and fitting results: (**a**) curves of average grain size over time; (**b**) curves fitted by grain growth index equation.

To specify the effects of grain refinement generated by the particles in three different situations, the corresponding grain average size curves are plotted in Figure 7.

In Figure 7a, the grain growth curve for the structure with elliptical particles (*f* = 10%, *a*_1_ = 4 μm, *b*_1_ = 1 μm) is located at the bottom of all curves, indicating that the average size of the corresponding grains at the same moment was the smallest, that is, the stronger the pinning effect of the particles on grain boundaries, the smaller the grain size, and the more obvious the effect of fine grain strengthening.

Figure 7b shows the fitted curve of the simulated grain growth curve according to the grain growth index formula, and the variation of the relevant parameters can be obtained as shown in Table 1. Here, the grain growth index equation is [32]:

(7) $$R_{ave}=kt^{1/m}+R_{0}$$

where *R*_ave_ is the average grain size of the matrix, which gradually increases with evolution until it stabilizes; *m* is the grain growth index; *R*_0_ is a constant; *k* is the time-dependent parameter, whose unit is related to *m*, and *t* is the time.

Table 1 shows that the corresponding *m* value of AZ31 Mg alloy matrix was 2.07 ≈ 2.0, and this result is in accordance with the law of non-conservative ordered vector field growth [32], which proves the reliability and feasibility of the model constructed in this study. Comparing the three cases when second-phase particles were included, it could be found that at an equal second phase particle volume fraction (*f* = 10%), the smaller the content of spherical particles, the larger the corresponding *m* value. This indicates that the hindering effect on grain growth gradually increased under the corresponding conditions, and it could also reflect that the fine grain strengthening effect of elliptical particles was better than that of spherical particles with an equal particle volume fraction.

**Figure 8:**
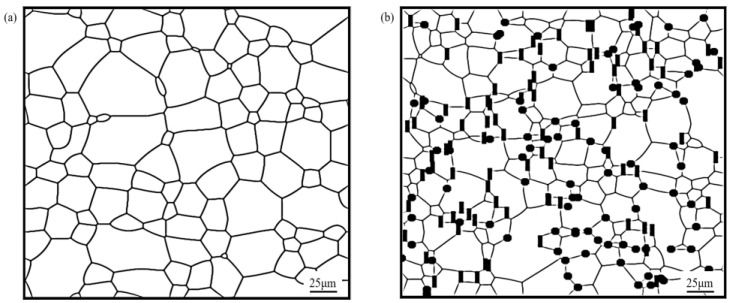

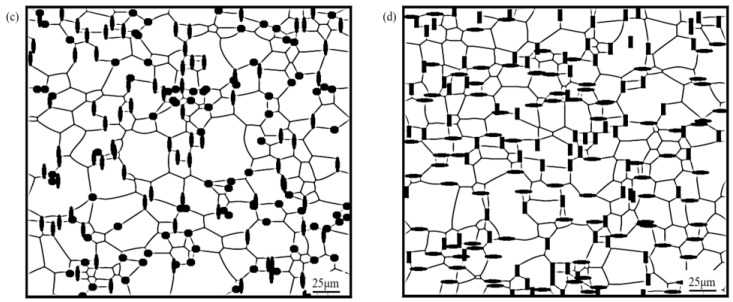
Microstructure of AZ31 magnesium alloy containing mixed shapes of particles at a 350 °C annealing temperature and 100 min annealing: (**a**) AZ31 magnesium alloy substrate; (**b**) spherical second phase particles *f*_1_ = 5%, *r* = 2 μm; rod-shaped second phase particles *f*_2_ = 5%, *a*_2_ = 2 μm, *b*_2_ = 6 μm; (**c**) spherical second phase particles *f*_1_ = 5%, *r* = 2 μm; elliptical second phase particles *f*_2_ = 5%, *a*_1_ = 4 μm, *b*_1_ = 1 μm; (**d**) elliptical second phase particles *f*_2_ = 5%, *a*_1_ = 4 μm, *b*_1_ = 1 μm; rod-shaped second phase particles *f*_1_ = 5%, *a*_2_ = 6 μm, *b*_2_ = 2 μm.

To further study the influence of mixing the shapes of particles on the grain growth of the alloy, a study was carried out mixing spherical, elliptical and rod-shaped particles with an equal particle volume fraction (*f* = 10%) two by two at an annealing temperature of 350 °C, with annealing for 100 min, to observe the microstructural evolution in each case, and the simulation results are shown in Figure 8.

Figure 8 shows the microstructure of magnesium alloy after the mixing (two by two) of spherical, elliptical and rod-shaped particles with an equal particle volume fraction. In terms of the distribution of particles, the distribution of mixed shaped particles was consistent with the distribution of single-shaped particles; that is, spherical particles tended towards trigonal grain boundary distribution, while elliptical and rod-shaped particles were distributed in the direction of the grain boundary.

**Figure 9:**
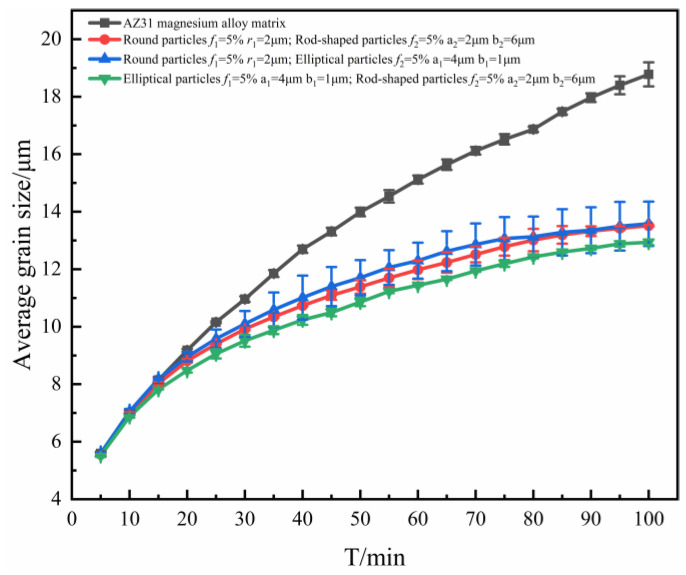
Average grain size of AZ31 magnesium alloy containing mixed second phase particles of different shapes with the same annealing time.

To specifically analyze the effects of mixing the shapes of particles on grain growth, the corresponding grain average size curves are plotted in Figure 9.

From Figure 9, it can be seen that the average grain size curves corresponding to the mixture of spherical and elliptical particles and the mixture of spherical and rod-shaped particles are within the error tolerance, and in good agreement. It was considered that the pegging effect of mixed particles on grain boundaries was the same in both cases, and the fine grain strengthening effect on grains was the same. It could be found that the average grain size curve corresponding to the mixture of elliptical and rod-shaped particles is significantly lower than the other two cases. It is also shown that elliptical and rod-shaped particles were better than spherical particles in terms of grain refinement effects at an equal particle volume fraction.

## 4. Conclusions

(1) The introduction of second phase particles had an obvious refining effect on the grain growth of AZ31 Mg alloy for fine grain strengthening;
(2) When the content of the particles was certain, the effect of spherical particles on grain refinement was the weakest. The elliptical particles and rod-shaped particles exhibited similar refinement effects within 100 min;
(3) The effect of the spatial arrangement direction of non-isometric elliptical particles on grain growth has been studied, and the results show that the spatial arrangement direction of the second phase particles had no significant effect on grain growth;
(4) According to this study of the effect of adding different shapes of mixed particles with equal particle volume fractions into the microstructure, the lower the proportion of circular particles, the better the refinement effect of the particles on the microstructure.

## Figures and Tables

**Table 1:** Variation of parameters obtained by fitting the grain growth index equation.

	*R*_ave_ = *kt*^1/*m*^ + *R*_0_			
	*k*	*R*_0_ (μm)	*m*	R^2^
AZ31 magnesium alloy matrix	1.89	1.29	2.07	0.99
spherical particles *f* = 10% *r* = 2 μm	13.16	−11.16	6.85	0.99
spherical particles *f*_1_ = 5% *r* = 2 μm; elliptical particles *f*_2_ = 5% a_1_ = 4 μm b_1_ = 1 μm	12.27	−9.55	8.12	0.99
elliptical particles *f* = 5% a_1_ = 4 μm b_1_ = 1 μm	22.73	−20.90	10.88	0.99

## Data Availability

Not applicable.
